# Untargeted metabolomics based on HS- SPME-GC-MS revealing the dynamic evolution of aroma components during cigar aging

**DOI:** 10.3389/fpls.2025.1657415

**Published:** 2025-09-15

**Authors:** Mingxuan Yang, Huina Zhou, Xi Hu, Xiangyu Liu, Jinshan Lei, Keyuan Ye, Ting Zhou, Jun Hu, Baojiang He

**Affiliations:** ^1^ Key Laboratory in Flavor and Fragrance Basic Research, Zhengzhou Tobacco Research Institute, China National Tobacco Corporation, Zhengzhou, China; ^2^ China Tobacco Gene Research Center, Zhengzhou Tobacco Research Institute, China National Tobacco Corporation, Zhengzhou, China; ^3^ Industrial Efficient Utilization of Domestic Cigar Tobacco Key Laboratory of Sichuan Province, Sichuan China Tobacco Industry Co., Ltd., Chengdu, China

**Keywords:** cigar volatilomics, rOAV, K-means clustering, KEGG pathway, aroma dynamic changes

## Abstract

Cigar aging plays a critical role in enhancing flavor complexity and overall quality of cigar. In this study, an untargeted metabolomics method based on headspace solid-phase microextraction coupled with gas chromatography–mass spectrometry (HS-SPME-GC-MS) was used to comprehensively profile volatile compounds and track their dynamic changes during aging. A total of 1,836 volatile compounds were identified, primarily comprising heterocyclics, terpenoids, ketones, and esters. Multivariate analyses, including principal component analysis (PCA) and orthogonal partial least squares discriminant analysis (OPLS-DA), segmented the aging process into four distinct stages, with most differential metabolites showing upregulated trends. Notably, terpenoids exhibited substantial increases in both diversity and abundance. KEGG pathway enrichment analysis highlighted significant involvement of sesquiterpenoid and triterpenoid biosynthesis pathways in cigar aging process. Analysis based on relative odor activity values (rOAV) indicated a progressive enhancement of fruity, floral, honey, woody, and sweet notes, while coffee, roasted, hay, burnt, and spicy aromas declined over time. By integrating rOAV data with K-means clustering analysis, 21 key aroma-active compounds were identified to be closely associated with the aroma changes during aging, including 14 consistently upregulated compounds (e.g., (E)-β-damascone, δ-cadinene) and 7 downregulated ones (e.g., 2-ethyl-3,5-dimethylpyrazine, 3-octen-2-one). These findings provide new insights into the metabolic basis of cigar aging and offer a scientific foundation for optimizing industrial aging processes.

## 1 Introduction

Cigars are premium tobacco products composed entirely of whole tobacco leaves—including the wrapper, binder, and filler—and are widely appreciated for their mellow flavor and rich aroma (Jiang et al., 2024; Yang et al., 2024). The production process involves multiple stages, including cultivation, curing, fermentation, rolling, and aging (Hu et al., 2022b; Si et al., 2023). Freshly rolled cigars often exhibit harshness and poor flavor harmony, thereby necessitating an aging stage to enhance overall sensory quality (Zhang et al., 2024a). Aging is typically conducted under controlled temperature and humidity conditions, which sustain the activity of endogenous enzymes and microorganisms. These enzymatic and microbial agents facilitate the degradation of irritating substances and promote the integration of aroma-related compounds, thereby improving the smoothness and complexity of the final product (Xue et al., 2023). The duration of aging depends on cigar type and market tiers, ranging from several weeks for mass-market products to multiple years for premium lines. As the saying goes, “30% of a cigar’s quality comes from the leaf, 70% from aging,” which underscores the critical role of post-production maturation (Zhu et al., 2025).

In recent years, advanced analytical techniques have been applied to explore the chemical transformations and mechanisms involved in cigar aging. Zhu et al. (2025) used HS-GC-IMS to analyze volatiles under varying aging conditions, identifying aging time as the most influential factor in aroma differentiation. Hu et al. (2023) combined sensory analysis, chemical profiling, and microbial sequencing to assess the impact of different aging media, finding that a “coffee” media significantly enhanced aroma complexity and altered the surface microbiota within 30 days. Xue et al. (2023) revealed how aging environments regulate microbial successions and the degradation of sugars and alkaloids, underscoring the synergy effect of microbials and chemical transformations in cigar flavor development. However, current research mainly focuses on short-term aging (<90 days) or specific aging conditions, with limited insights into the long-term dynamics of aroma compounds, the identification of key volatiles, and their functional roles during extended aging.

Cigar flavor is a key quality indicator, reflecting consumer preferences and production evaluation. It depends on the composition and abundance of volatile compounds. HS-SPME-GC-MS is widely recognized as a preferred technique for analyzing volatiles in complex plant matrices due to its solvent-free operation, simplicity, and high sensitivity (Baky et al., 2024). The relative odor activity value (rOAV), calculated as the ratio of compound concentration to its odor threshold, is commonly used to quantify each volatile’s contribution to overall aroma (Sun et al., 2022). Compounds with rOAV ≥ 1 are considered key aroma contributors, while those with values ≥10 are regarded as having a pronounced sensory contribution (Xiao et al., 2022; Gou et al., 2023). Liang et al. (2024) applied HS-SPME-GC-MS in conjunction with metabolomics and rOAV analysis to identify key aroma compounds in Liubao tea, revealing the variation patterns of these compounds during fermentation and their influence on the flavor notes of Liubao tea. Even though this approach has been widely applied in tea, coffee, and other botanical systems (Mourão et al., 2023; Gu et al., 2025; Zhang et al., 2025), it has been scarcely explored in the context of cigar aging.

This study utilized HS-SPME-GC-MS combining with rOAV analysis to investigate the temporal dynamics of volatile metabolites during long-term cigar aging, with a specific focus on identifying key aroma-active compounds and elucidating their formation mechanisms. This work aims to advance the understanding of aroma evolution during cigar aging.

## 2 Materials and methods

### 2.1 Samples and reagents

Cigar samples were manufactured by the Great Wall Cigar Factory (China Tobacco Sichuan Industrial Co., Ltd.) using a fixed commercial formula, with the wrapper sourced from Ecuador, the binder from Indonesia, and the filler comprising a mixture of cigar tobacco leaves from Sichuan (China) and the Dominican Republic. All cigars were hand-rolled by the same experienced roller and immediately stored at –20°C for two days to prevent insect damage. Aging was performed in Spanish cedar cabinets under controlled conditions (60% ± 2% relative humidity, 20°C ± 2°C). Samples were collected at 0 (S0), 1 (S1), 2 (S2), 4 (S3), 17 (S4), and 18 (S5) months. At each collection, six cigars were randomly selected as replication for HS-SPME-GC-MS analysis. All samples were stored at –80°C in sterile bags until further analysis. To monitor analytical stability, quality control (QC) was inserted every ten samples during instrumental analysis. The QC was generated by pooling equal volumes from all experimental samples.

Sodium chloride (NaCl) was obtained from Merck (USA). The C7–C40 alkane standard and the internal standard 3-Hexanone-2,2,4,4-d_4_ were purchased from Sigma-Aldrich (Shanghai, China). Ultrapure water was prepared using a Smart2Pure system (Thermo Fisher Scientific, USA).

### 2.2 Sample pretreatment

Frozen cigar samples were removed from –80°C storage, sectioned, and ground under liquid nitrogen. 500 mg of the resulting powder was accurately weighed on an MS105DU analytical balance (Mettler Toledo, Zurich, Switzerland) and transferred into a 20 mL headspace vial (Agilent, Palo Alto, CA, USA). Subsequently, 2 mL of saturated NaCl solution and 10 μL of internal standard solution (3-Hexanone-2,2,4,4-d_4_, 10 μg/mL) were added with a pipette (Eppendorf, Hamburg, Germany).

### 2.3 Extraction and identification of volatile metabolites

Volatiles were extracted using HS-SPME according to the method described by Chen et al. (2025). After 5 min of incubation at 60°C, a 120 μm DVB/CWR/PDMS fiber (SPME Arrow, Agilent) was inserted into the vial for 15 min of headspace extraction. The fiber was then desorbed in the injector at 250°C for 300 s.

GC-MS analysis was conducted on an Agilent 8890 gas chromatograph coupled with a 7000D triple quadrupole mass spectrometer, equipped with a DB-5MS capillary column (30 m × 0.25 mm × 0.25 μm). Helium (≥99.999%) was used as the carrier gas at a constant flow rate of 1.2 mL/min. Injection was performed in splitless mode at 250°C, with a solvent delay of 3.5 min. The oven program was: 40°C (3.5 min) → 100°C at 10°C/min → 180°C at 7°C/min → 280°C at 25°C/min (5 min hold). The MS was operated in selected ion monitoring (SIM) mode with electron impact (EI) ionization at 70 eV. The ion source, quadrupole, and transfer line temperatures were set at 230°C, 150°C, and 280°C, respectively. For the qualitative identification of each compound, one quantitative and two to three qualitative ions were selected and monitored sequentially according to their elution times. The compound was considered successfully identified when the observed retention time (RT) was consistent with the entry in our local database (MWGC database) (Yuan et al., 2022), and all pre-selected ions were still detected after background signal subtraction.

Semi-quantification was performed using 3-Hexanone-2,2,4,4-d_4_ as the internal standard. The concentration of compound $X_{i}$ (μg/g) was calculated using the following equation:


$$X_{i}=\frac{V_{s}*C_{s}}{M}*\frac{I_{i}}{I_{s}}*10^{-3}$$

Where: $V_{s}$ is the volume of the internal standard (μL), $C_{s}$ is its concentration (μg/mL), M is the sample mass (0.5 g), $I_{i}$ is the peak area of compound *i*, and $I_{s}$ is the peak area of the internal standard.

### 2.4 Calculation of relative odor activity value

Key aroma compounds were screened based on their relative odor activity value (rOAV), which was calculated as the ratio of each compound’s concentration to its odor threshold. Odor descriptors and threshold data were obtained from the Good Scents Company (http://www.thegoodscentscompany.com), the Perflavory database (http://perflavory.com), the LRI & Odor Database – Odor Data (http://www.odour.org.uk/odour/index.html), and the Food Flavor Lab (http://foodflavorlab.cn/#/home). The rOAV for each compound was calculated as follows, according to the method described by Peng et al. (2024):


$$rOAV_{i}=\frac{X_{i}}{T_{i}}$$

where $X_{i}$ is the concentration (μg/g) and $T_{i}$ is the odor threshold (μg/g) of compound *i*.

### 2.5 Statistical analysis

Statistical analyses were performed using Python and R. One-way ANOVA followed by Tukey’s *post hoc* test was conducted in Python (statsmodels, v0.14.1), with statistical significance set at *p*< 0.05. Groups labeled with the same letter were considered not significantly different. PCA was performed to discriminate the volatiles changes upon cigar aging according to Maurya et al. (2018), and was computed using the ‘prcomp’ function in R. The Pearson correlation coefficients were computed using the ‘cor’ functions. Heatmaps were visualized using the ‘ComplexHeatmap’ package. OPLS-DA was performed using the ‘MetaboAnalystR’ package, and differential metabolites were identified based on variable importance in projection (VIP) > 1, absolute log_2_ fold change (|log_2_FC|) > 1.0, and *p*< 0.01. Unsupervised clustering was performed using the ‘kmeans’ function on unit variance (UV)-scaled data, and clustering trends were visualized using the ‘ggplot2’ package. KEGG annotation of differential metabolites was conducted using the KEGG Compound Database (http://www.kegg.jp/kegg/compound/) and KEGG Pathway Database (http://www.kegg.jp/kegg/pathway.html). Subsequent pathway enrichment analysis was performed using metabolite set enrichment analysis (MSEA) with hypergeometric testing to assess significance.

## 3 Results

### 3.1 Overview of volatile metabolites changes during cigar aging

**Figure 1:**
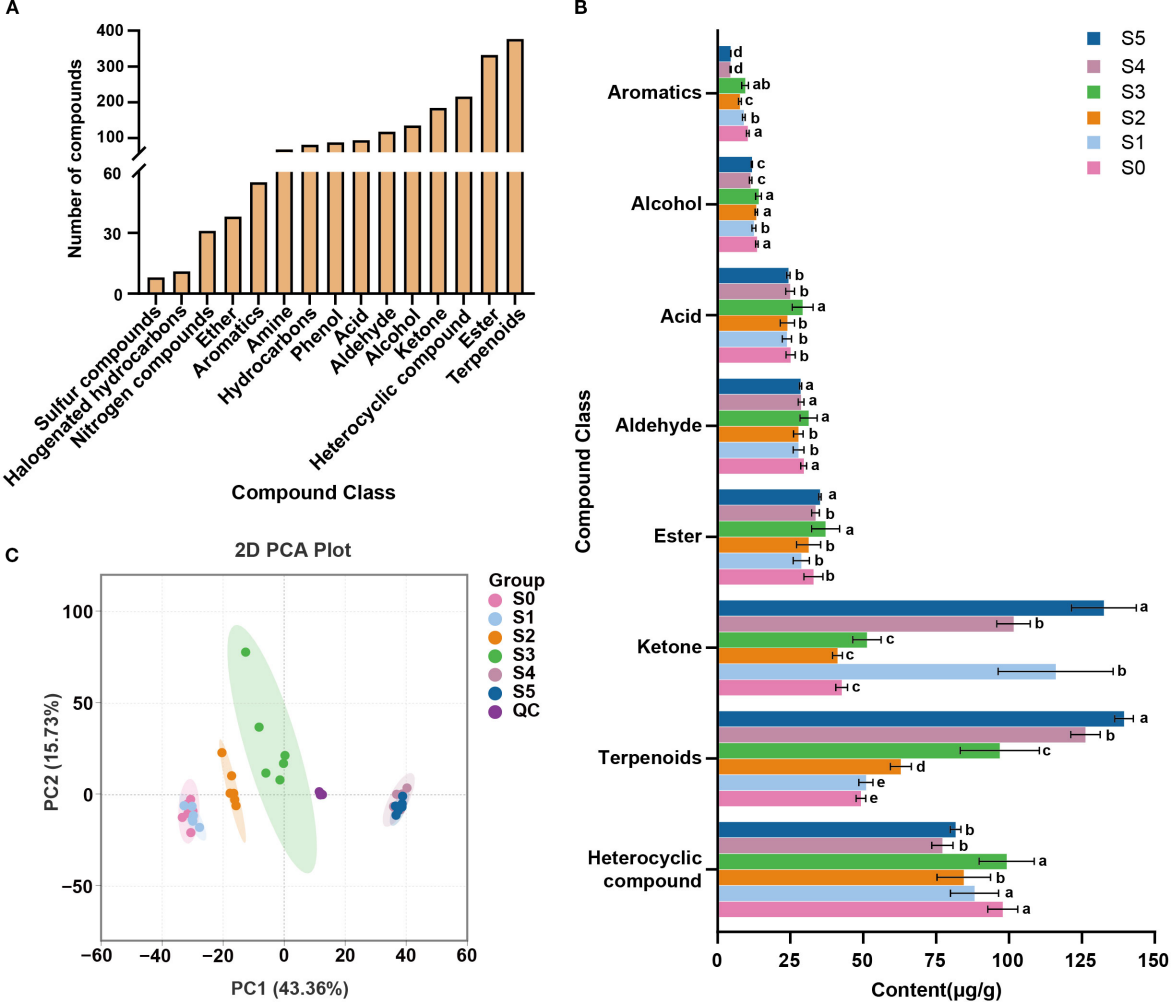
Volatile compounds classification and their compositional changes during cigar aging. **(A)** The number of volatile metabolites detected in cigar samples; **(B)** Changes in volatile metabolites during cigar aging. Different letters indicate significant differences (*p*< 0.05) among different sampling times; **(C)** PCA analysis of cigar samples from 18 months aging process based on volatile metabolites.

A total of 1,836 volatile metabolites were identified across all sampling time points (**Figure 1A**; **Supplementary Table S1**), including 377 terpenoids, 332 esters, 216 heterocyclics, 184 ketones, and other compound classes, with terpenoids being the most abundant compounds. As shown in **Figure 1B**, heterocyclics, terpenoids, ketones, and esters were the most abundant and exhibited pronounced temporal variation. Notably, terpenoid content increased from 49.18 μg/g at S0 to 139.5 μg/g at S5.

Principal component analysis (PCA) was used to visualize the temporal distribution of all samples and distinguish aging stages. In the two-dimensional PCA score plot (**Figure 1C**), the first two principal components (PC1 and PC2) explained 43.36% and 15.73% of the variance, respectively, accounting for a cumulative 59.09% explanation. Cigar samples from different time points were clearly separated, and tightly clustering of QC samples demonstrated high analytical reproducibility. Along PC1, samples exhibited a time-ordered distribution. Overlap was observed between S0 and S1 (months 0 and 1), and between S4 and S5 (months 17 and 18), indicating similar volatile profiles at those samples. Based on these results, the aging process was divided into four stages: G1 (S0–S1), G2 (S2), G3 (S3), and G4 (S4–S5).

### 3.2 Analyses of differential volatile metabolites across cigar aging stages

OPLS-DA was used to examine changes of volatile metabolites among different cigar aging stages. Three pairwise comparison models were constructed: G1 vs G2, G1 vs G3, and G1 vs G4. Score plots (**Supplementary Figures S1A–C**) showed clear separation between groups, and 200-permutation tests validated the models. All models yielded R²Y > 0.99 and Q² > 0.98 (**Supplementary Figures S1D–F**), indicating the strong explanatory power and predictive accuracy.

**Figure 2:**
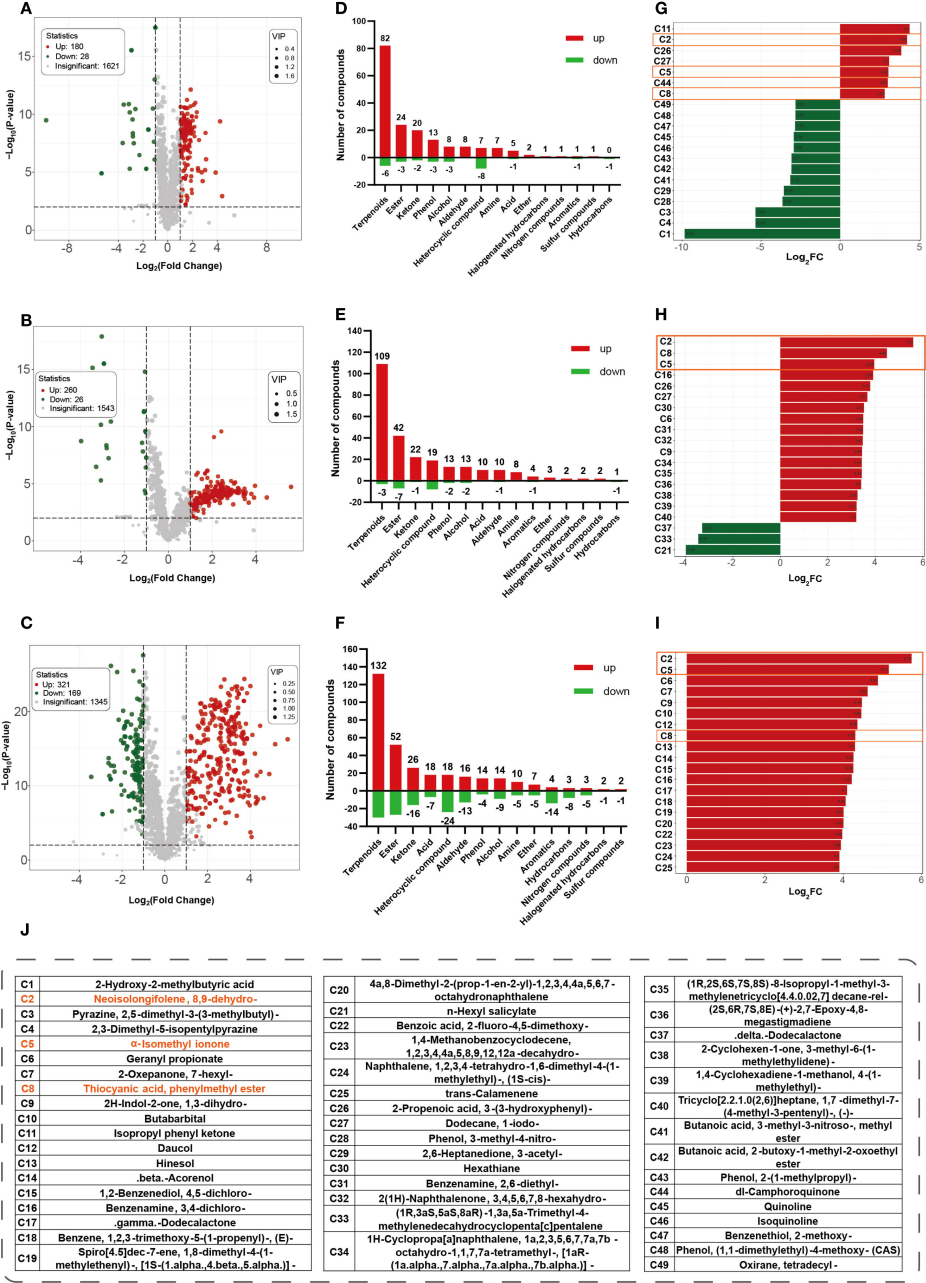
Differential metabolites analysis at different aging stages compared with G1. Volcano plots of G1 vs G2 **(A)**, G1 vs G3 **(B)**, and G1 vs G4 **(C)**, (x-axis indicates fold change magnitude, y-axis represents statistical significance. Red dots indicate significantly upregulated metabolites, green dots indicate downregulated ones, and gray dots indicate non-significant differences). Bidirectional bar charts showing the number of up- and downregulated metabolites (numbers on the y-axis and bars indicate the count) for G1 vs G2 **(D)**, G1 vs G3 **(E)**, G1 vs G4 **(F)**. Top 20 metabolites with the highest |log_2_FC| values in comparison group of G1 vs G2 **(G)**, G1 vs G3 **(H)**, G1 vs G4 **(I)**. Red indicates upregulation, green indicates downregulation. Compound names are shown as codes, and compounds common to all three comparisons are outlined in solid orange boxes. **(J)** compounds names for those codes in **(G-I)**.

Differential volatile metabolites were identified based on combined selection criteria: VIP ≥ 1, |log_2_FC| ≥ 1, and *p*< 0.01 (**Supplementary Tables S2**-**S4**). Volcano plots (**Figures 2A–C**) visualized expression differences relative to G1, revealing 180, 260, and 321 upregulated volatiles, and 28, 26, and 169 downregulated volatiles in G2, G3, and G4, respectively. Terpenoids, esters, and ketones showed the largest increases and continued to accumulate with aging (**Figures 2D–F**). The top 20 metabolites ranked by |log_2_FC| in each comparison were pooled, resulting in 49 unique compounds (**Figures 2G–I**). The proportion of upregulated metabolites among these top-ranking compounds increased with aging time, reaching to 100% in the G1_vs_G4 group. Notably, 8,9-dehydro-neo-isolongifolene (C2), α-isomethyl ionone (C5), and benzyl thiocyanate (C8) were common to all three comparisons (highlighted in orange boxes); the first two exhibited progressively increasing fold changes and continuous accumulation during cigar aging.

**Figure 3:**
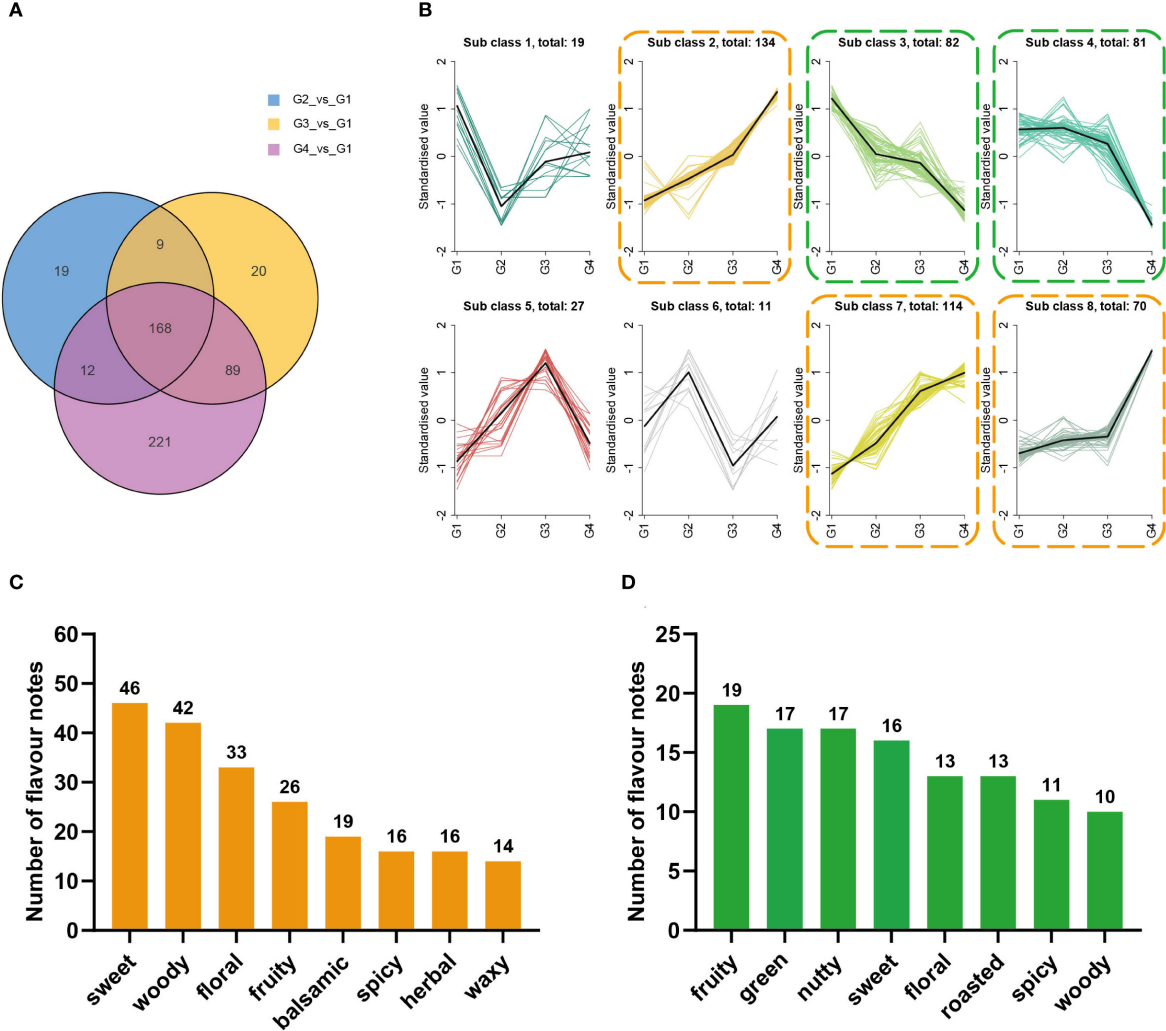
K-means clustering analysis and sensory flavor attribute analyses for differential volatile metabolites related to cigar aging. **(A)** Venn diagram of differential volatile metabolites for G1 vs G2, G1 vs G3, and G1 vs G4. **(B)** K-means clustering analysis of differential metabolites in cigar samples. The x-axis represents sample groups, and the y-axis indicates the normalized relative abundance of metabolites.; the number after “total: “ indicates the total number of metabolites in that subclass. **(C)** Sensory flavor attributes for differential volatile compounds in sub classes 2, 7, and 8 [highlighted with orange dashed boxes in panel **(B)**]. The x-axis represents the annotated sensory flavor attributes, and the y-axis indicates the number of differential metabolites associated with each attribute. **(D)** Sensory flavor attributes for differential volatile compounds in sub classes 3 and 4 [highlighted with green dashed boxes in panel **(B)**]. The x-axis represents the annotated sensory flavor attributes, and the y-axis indicates the number of differential metabolites associated with each attribute.

Totally, 538 differential volatiles were identified, and 168 were shared across the comparisons (**Figure 3A**). K-means clustering (**Supplementary Table S5**) revealed eight expression patterns. Compounds in Sub class 2, Sub class 7, and Sub class 8 (**Figure 3B**, orange) were predominantly upregulated, while those in Sub class 3 and Sub class 4 (green) were mainly downregulated. Sensory annotations such as “sweet,” “woody,” “floral,” and “fruity”, were mostly annotated in upregulated group (**Figure 3C**) compared with the downregulated group (**Figure 3D**).

### 3.3 Pathway enrichment analysis of differential volatile metabolites during cigar aging

**Figure 4:**
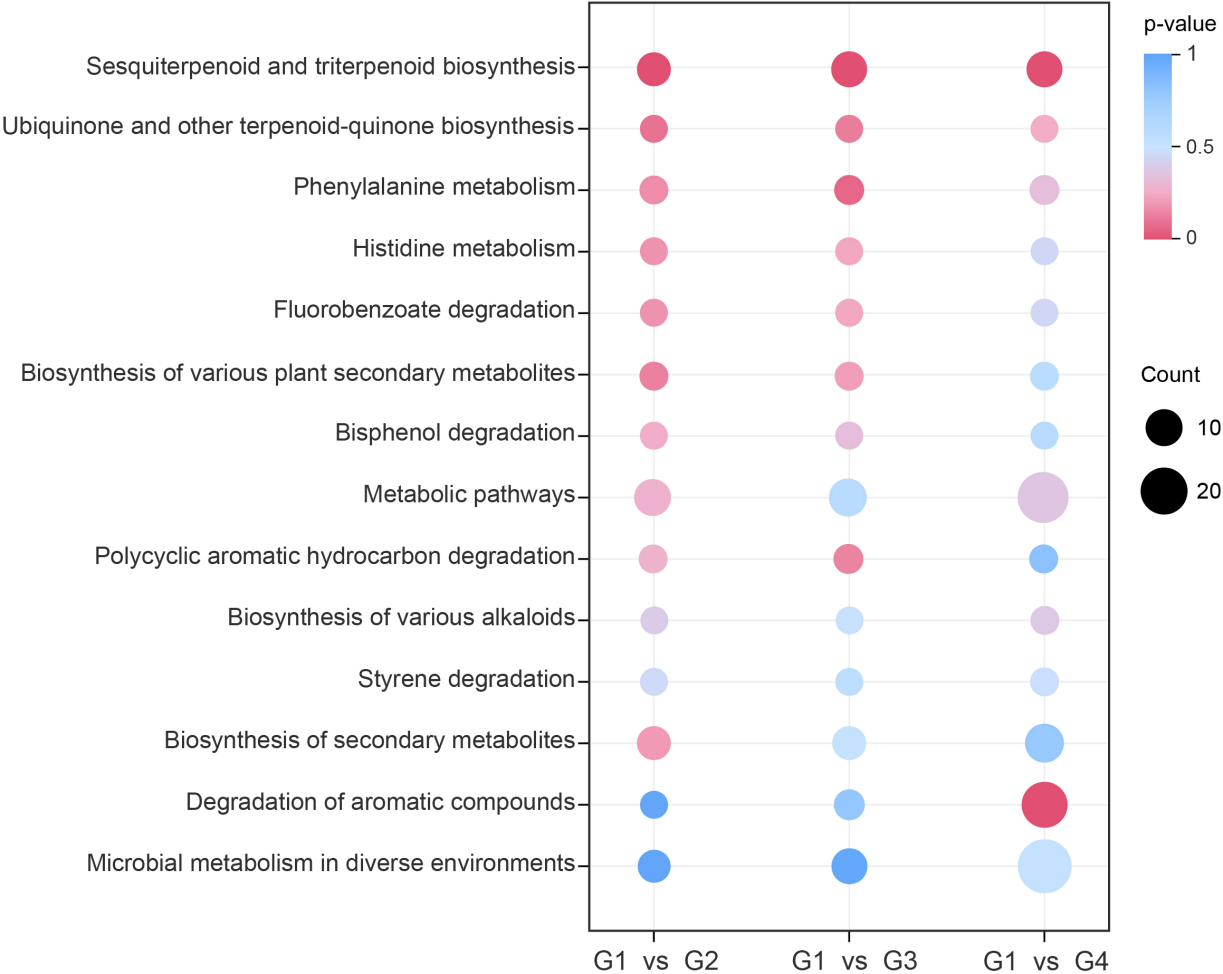
KEGG pathway enrichment of differential volatile metabolites. The y-axis displays the names of enriched pathways sorted in ascending order by p-value, and the x-axis represents the comparison groups. The size of each dot indicates the number of differential volatile metabolites enriched in the corresponding pathway, while the color gradient reflects the p-value magnitude.

To investigate the possible biological process involved in cigar aging, KEGG-based pathway enrichment analysis was performed for comparisons of G1 vs G2, G1 vs G3, and G1 vs G4. A total of 14, 18, and 30 metabolic pathways were significantly enriched in the three comparisons (**Supplementary Tables S6**-**S8**), respectively, with 14 pathways shared across all groups (**Figure 4**). Notably, the sesquiterpenoid and triterpenoid biosynthesis pathway was significantly enriched (*p*< 0.05) in all comparisons, indicating its pivotal role in the dynamic remodeling of volatile profiles throughout the cigar aging process.

**Figure 5:**
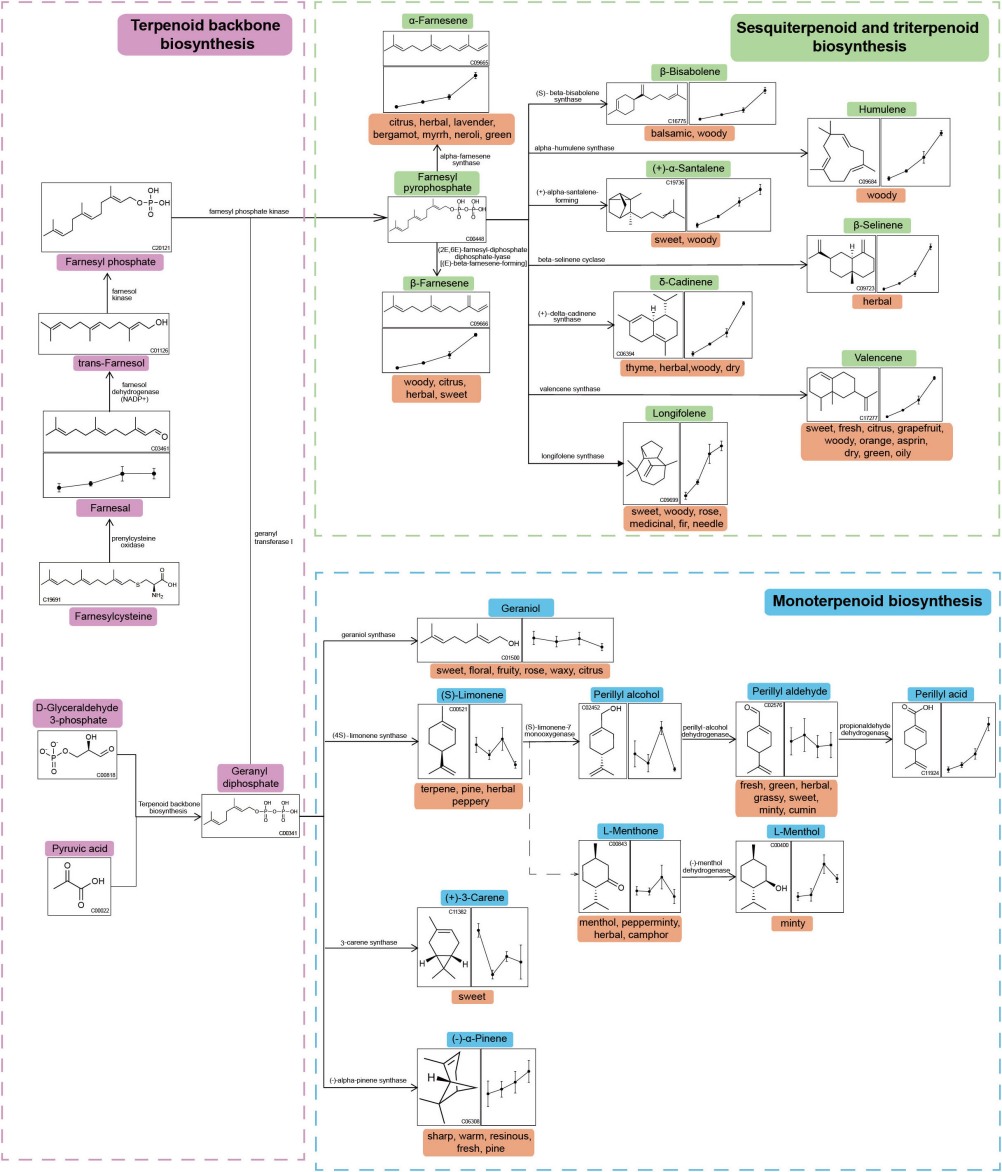
Metabolic network of terpenoid compounds during cigar aging. The line graphs from left to right illustrate the changes in metabolite abundance from G1 to G4. The odor attributes associated with each metabolite are noted beneath their names.

Terpenoids were predominant not only in the total pool of volatiles but also among the differentially expressed metabolites. The sesquiterpenoid and triterpenoid biosynthesis pathway shares a common upstream origin with monoterpenoid biosynthesis, both being downstream branches of the terpenoid backbone biosynthesis pathway. To further elucidate terpenoid metabolic dynamics, a detailed metabolic pathway diagram was constructed and annotated based on KEGG data (**Figure 5**). Several sesquiterpenes, including δ-cadinene, β-selinene, valencene, longifolene, β-farnesene, humulene, α-farnesene, β-bisabolene, and (+)-α-Santalene, exhibited a clear trend of continuous accumulation during aging. Farnesal, a key upstream precursor for the biosynthesis of farnesyl pyrophosphate (FPP), also showed a gradual increase in abundance.

In contrast, several monoterpenes such as linalool and geraniol exhibited declining or fluctuating trends over time. However, a few compounds within the monoterpenoid pathway—including perillaldehyde and α-pinene—displayed upward trends, suggesting selective accumulation among specific structural classes. These observations underscore the distinct metabolic trajectories of mono- and sesquiterpenes during long-term cigar aging.

### 3.4 Sensory analysis based on rOAV and key aroma compounds selection

To identify key aroma contributors, all volatiles were evaluated using rOAV (**Supplementary Table S9**). Ketones, terpenoids, esters, and heterocyclics had the highest total rOAV values (**Supplementary Figure S2**), all showing significant changes across aging stages. The total rOAV of terpenoids increased steadily from G1 to G4, underscoring their growing impact on cigar aroma during aging.

**Figure 6:**
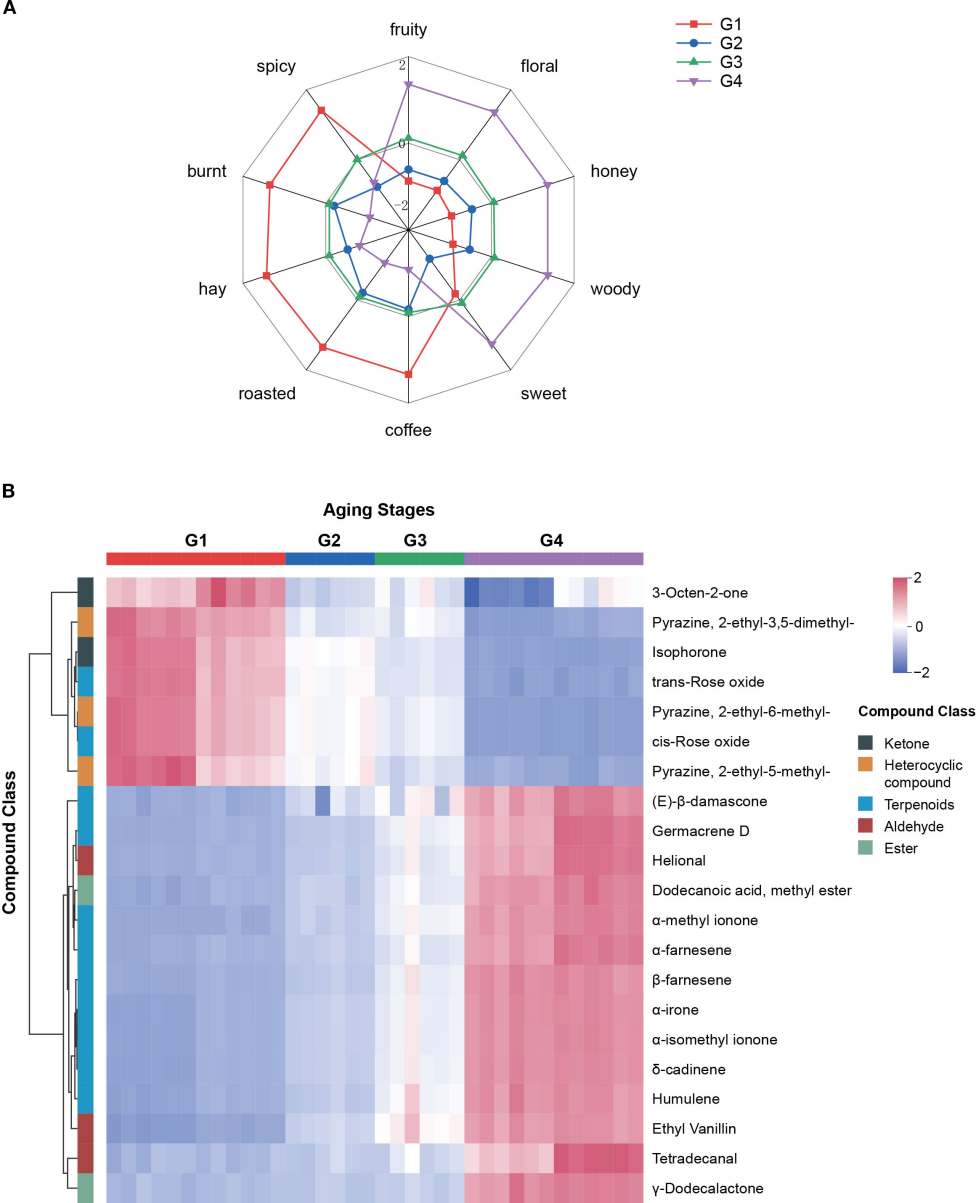
Changes in sensory flavor attributes during cigar aging **(A)** and heatmap of 21 key aroma compounds **(B)**. In radar chart, showing the ten aroma attributes of cigars, the coordinates represent the UV-standardized sum of rOAV values corresponding to each aroma attribute.

A radar chart was constructed according to the method of Guo et al. (2021) to visualize changes in sensory attributes across the aging process. The chart’s coordinates represent the UV-standardized rOAV values, which were calculated as the average of rOAV values for compounds within the same aroma category. The results (**Figure 6A**) showed that fruity, floral, honey, woody, and sweet attributes intensified over time, while coffee, roasted, hay, burnt, and spicy notes gradually declined. Notably, the sweet attribute showed a temporary decrease during stage G2, followed by a substantial increase in G3 and G4. This fluctuation may be attributed to the dynamic accumulation of furanones, particularly dihydro-2-methyl-3(2H)-furanone and 4-hydroxy-2,5-dimethyl-3(2H)-furanone (HDMF), both of which showed high odor activity. Their rOAV changes, which were lower in G2 but increased in later stages, may have significantly contributed to the enhancement of the sweet aroma (**Supplementary Table S9**).

Based on K-means clustering (**Figure 3B**), from those continuously increased or decreased compounds, 21 key aroma-active compounds were defined as key contributors (**Supplementary Table S10**) with rOAV>1 in either G1 or G4 stage, including 14 consistently upregulated and 7 consistently downregulated across aging stages. Among the upregulated compounds, terpenoids were the most prevalent (9 out of 14), followed by aldehydes. The fruity, floral, and tobacco-scented compound (E)-β-damascone exhibited the highest rOAV. Other terpenoids with strong accumulation trends included δ-cadinene, α-methyl ionone, α-irone, and α-isomethyl ionone, all of which contributed to the intensification of fruity, floral, honey, and woody notes during aging (**Figure 6A**). Heterocyclics were the dominant class among the seven downregulated compounds. Notably, 2-ethyl-3,5-dimethylpyrazine, a roasted- and coffee-scented compound widely found in baked goods, tea, coffee, and alcoholic beverages, and 3-octen-2-one, a hay-scented ketone, both exhibited high initial rOAV that declined substantially during aging. Their decreasing abundance partly explains the fading of coffee, roasted, hay, burnt, and spicy sensory attributes over time.

## 4 Discussion

Based on an 18-month aging experiment, HS-SPME-GC-MS analysis was employed to reveal the substantial impact of long-term aging on the volatile metabolite profile of cigars. PCA results segmented the aging progression into four stages (G1–G4). Terpenoid compounds began accumulating during G1 to G2 and showed a continuous upward trend, reaching their peak abundance at G4. This trend aligns with findings from tea and beer aging, where terpenoids also exhibit dynamic accumulation (Wang et al., 2008; Ma et al., 2023).

OPLS-DA and K-means clustering revealed stage-dependent changes in volatile metabolites during cigar aging. The number of significantly upregulated compounds increased from 180 in G2 to 321 in G4, while the number of downregulated compounds remained comparatively low throughout the aging process (**Figures 2A–C**). Venn analysis identified 221 unique volatiles in the G4 vs G1 comparison, substantially exceeding the numbers found in earlier stages (**Figure 3A**). Among the 538 differential metabolites, 481 (89.4%) exhibited clear trends of up- or downregulation based on K-means clustering (**Figure 3B**), highlighting the dynamics of volatile changes over time. Most upregulated compounds were terpenoids, esters, and ketones, with terpenoids exhibiting the most pronounced accumulation trend (**Figures 2D–F**). This pattern parallels the accumulation of volatiles observed during cigar leaf fermentation (Fan et al., 2023), suggesting the existence of shared metabolic trajectories. Overall, long-term aging induced substantial shifts in volatile composition, particularly through sustained terpenoid accumulation, which likely contributes to aroma maturation and flavor complexity.

Pathway enrichment analysis of differential volatile metabolites based on the KEGG database revealed that the sesquiterpenoid and triterpenoid biosynthesis pathway was significantly enriched (*p*< 0.05) in all three comparisons—G1_vs_G2, G1_vs_G3, and G1_vs_G4—indicating sustained metabolic activity of this pathway during long-term cigar aging. Terpenoid biosynthesis pathways, including those for sesquiterpenoids and triterpenoids, originate from the fundamental precursors isopentenyl pyrophosphate (IPP) and dimethylallyl pyrophosphate (DMAPP) (Huo et al., 2022). These precursors are converted by isopentenyl transferases into intermediates such as geranyl pyrophosphate (GPP) and farnesyl pyrophosphate (FPP) (Luo et al., 2015). Among these, FPP functions as a crucial metabolic node and serves as the direct precursor for sesquiterpenoid and triterpenoid synthesis. Through various terpene synthases, FPP is transformed into woody-scented sesquiterpenes such as δ-cadinene, β-selinene, valencene, longifolene, β-farnesene, humulene, and α-farnesene (Sallaud et al., 2009; Moses et al., 2013; Wang et al., 2024). Similarly, Naziz et al. (2019) observed that activation of sesquiterpenoid and triterpenoid biosynthetic pathways was closely associated with woody aroma formation in *Aquilaria* spp. under stress conditions. Combined with our results, it was suggested that sesquiterpenoid and triterpenoid biosynthetic pathways played a central role in the biosynthesis of woody volatiles across plant systems.

Additionally, farnesal—the upstream precursor of FPP—exhibited a sustained upregulation trend during aging (**Figure 5**), in contrast to the decreasing pattern reported by Fan et al. (2023) during cigar leaf fermentation. This divergence suggests that accumulation of this intermediate may be highly stage-dependent. Given the critical role of downstream terpenoids in aroma formation, future studies could employ isotope tracing and other techniques to elucidate the biosynthesis and transformation pathways of farnesal in the context of cigar aging.

Moreover, the phenylalanine metabolism pathway was also enriched throughout aging. As a key route for the biosynthesis of aromatic compounds, this pathway provides important intermediates that influence cigar aroma. Zhang et al. (2024b) reported that phenylacetaldehyde, a floral-scented compound, accumulated during cigar leaf fermentation and was associated with elevated expression of the phenylalanine metabolism pathway. Furthermore, phenylalanine can be converted to cinnamic acid by phenylalanine ammonia-lyase (PAL), subsequently leading to the formation of floral and fruity volatiles such as benzaldehyde and benzyl alcohol (Skaliter et al., 2022). Song et al. (2024) showed that inoculating cigar tobacco leaves with *Bacillus altitudinis* during fermentation significantly enhanced the expression of enzymes involved in phenylalanine metabolism, resulting in a 43% increase in total aroma compounds compared to natural fermentation. These findings indicate that microbial community structure can affect aroma generation by modulating key metabolic pathways. This suggests that targeted microbial interventions may be promising for optimizing the aging process and steering the synthesis of desirable aroma compounds.

The dynamic evolution of aroma compounds plays a central role in shaping the sensory characteristics of cigars. Based on rOAV analysis, changes in the concentrations of compounds with distinct sensory notes may contribute to the transition in flavor from sharp and spicy in freshly rolled cigars to a richer, sweeter, and more balanced profile after extended aging (Zhu et al., 2025). Through K-means clustering and rOAV screening, 21 key aroma compounds were identified, including 14 that consistently increased and 7 that consistently decreased over time (**Figure 6B**; **Supplementary Table S10**). Among them, (E)-β-damascone and δ-cadinene were particularly prominent. (E)-β-damascone, known for its sweet-rosy and fruity characteristics, is a representative product of carotenoid degradation and a crucial contributor to tobacco aroma (Ma et al., 2020). It is naturally present in various processed plant products, such as tea (Zheng et al., 2016) and tobacco. In a relevant study, Ning et al. (2023) found that fermenting tobacco with specific strains (*Bacillus subtilis* B1 and *Cytobacillus oceanisediminis* C4) could significantly increase the content of (E)-β-damascone, enhancing the tobacco’s fruity and floral notes and demonstrating the important role of its elevated content in improving sensory quality. It acts synergistically with megastigma-3,5-dienone, solanone, and farnesyl acetone to shape the typical scent of tobacco (Popova et al., 2019). Despite its low concentration, it’s extremely lower odor threshold enables it to strongly enhance sweet and floral aromas in cigars (Hu et al., 2022c). δ-cadinene, a sesquiterpene with dry woody and slightly spicy notes, is a dominant terpene in the essential oils of *Chromolaena odorata*, *Aglaia odorata*, and *Juniperus* spp (Semerdjieva et al., 2024). It is widely used in fragrances, perfumes, and tobacco products, and is primarily found in well-aged or premium cigars, rarely appearing in fresh or early fermented cigar tobacco leaves (Hu et al., 2022a; Yang et al., 2024). Its accumulation during cigar aging in this study suggests a key role in the development of deep woody aroma.

Additionally, two furanones—dihydro-2-methyl-3(2H)-furanone and 4-hydroxy-2,5-dimethyl-3(2H)-furanone (HDMF)—showed a temporary decline in stage G2 followed by significant increases in G3 and G4 (**Supplementary Table S9**). Dihydro-2-methyl-3(2H)-furanone is typically formed via Maillard reactions in tobacco and contributes to a fuller smoke body, while HDMF, naturally present in strawberries and pineapples (Raab et al., 2006; Schwab, 2013), is a potent flavor enhancer with a very low threshold and is widely used in tobacco flavoring. As aging progresses, reducing sugars tend to accumulate in cigars (Xue et al., 2023), providing substrates for the slow but continuous Maillard reactions. This chemical pathway, involving the reaction of reducing sugars and amino acids, is the well-established mechanism for producing these caramel-like furanones in numerous thermally processed or aged products, including aged red wine, cheese, and soy sauce (Schwab, 2013; Wu et al., 2024). Specifically, research on the yeast *Zygosaccharomyces rouxii* has revealed a sophisticated chemo-enzymatic pathway where a sugar derivative (D-fructose-1,6-bisphosphate) is first chemically converted to a key intermediate, which is then enzymatically reduced by yeast enzymes to form the final HDMF, a compound noted for its attractive, sweet-caramel flavor (Hauck et al., 2003). In cigars, sweetness helps balance harshness and bitterness, enhancing smoothness and palatability, while woody notes contribute warmth and complexity (Ganz et al., 2022). A study of American cigar consumers revealed strong preferences for sweet and woody flavor attributes (Delnevo et al., 2015). The observed increase in volatile compounds related to these aromas during aging suggests a favorable impact on cigar quality. Collectively, the identification and temporal patterns of these key aroma compounds provide a chemical basis for optimizing aging practices and targeted aroma modulation. These findings indicate that adjusting aging duration or precursor availability may effectively enhance cigar flavor quality.

## 5 Conclusion

Based on an Untargeted metabolomics strategy using HS-SPME-GC-MS, this study systematically characterized the dynamic changes in volatile aroma compounds during long-term cigar aging. The results revealed that with increasing aging time, the volatile metabolite profile of cigars was significantly reshaped, and these differences accumulated over time, with terpenoids showing a continuous enrichment trend. KEGG enrichment analysis indicated that the sesquiterpenoid and triterpenoid biosynthesis pathway (*p*< 0.05) was significantly enriched across all comparison groups, and the farnesyl pyrophosphate (FPP)-centered metabolic network was important for the accumulation of woody-scented sesquiterpenes such as δ-cadinene, β-selinene, valencene, longifolene, β-farnesene, humulene, and α-farnesene. Relative odor activity value (rOAV) analysis further revealed that aging enhanced fruity, floral, honey, woody, and sweet aroma attributes while weakening coffee, roasted, hay, burnt, and spicy characteristics. A total of 21 key aroma compounds—including (E)-β-damascone, δ-cadinene, 2-ethyl-3,5-dimethylpyrazine, and 3-octen-2-one—were screened out based on rOAV screening and K-means clustering, providing a preliminary explanation for these sensory attribute transitions. Future studies are recommended to focus on the regulatory mechanisms of key enzymes and the biosynthesis of FPP in the sesquiterpenoid pathway, aiming to achieve precise modulation of cigar aroma and process optimization.

## Data Availability

The data presented in the study are deposited in the Plant Metabolomics Database (PMDB) and are publicly available at http://www.pmdb.org.cn/cigarMSdata.
