# Supplementary material for: Untargeted metabolomics based on HS- SPME-GC-MS revealing the dynamic evolution of aroma components during cigar aging
Source: Front Plant Sci. 2025 Sep 15;16:1657415. doi: 10.3389/fpls.2025.1657415 (PMC12477199; doi:10.3389/fpls.2025.1657415)
Supplement: Supplementary file 11 [file Supplementaryfile1.docx]

Supplementary Material

# Supplementary Figures

**
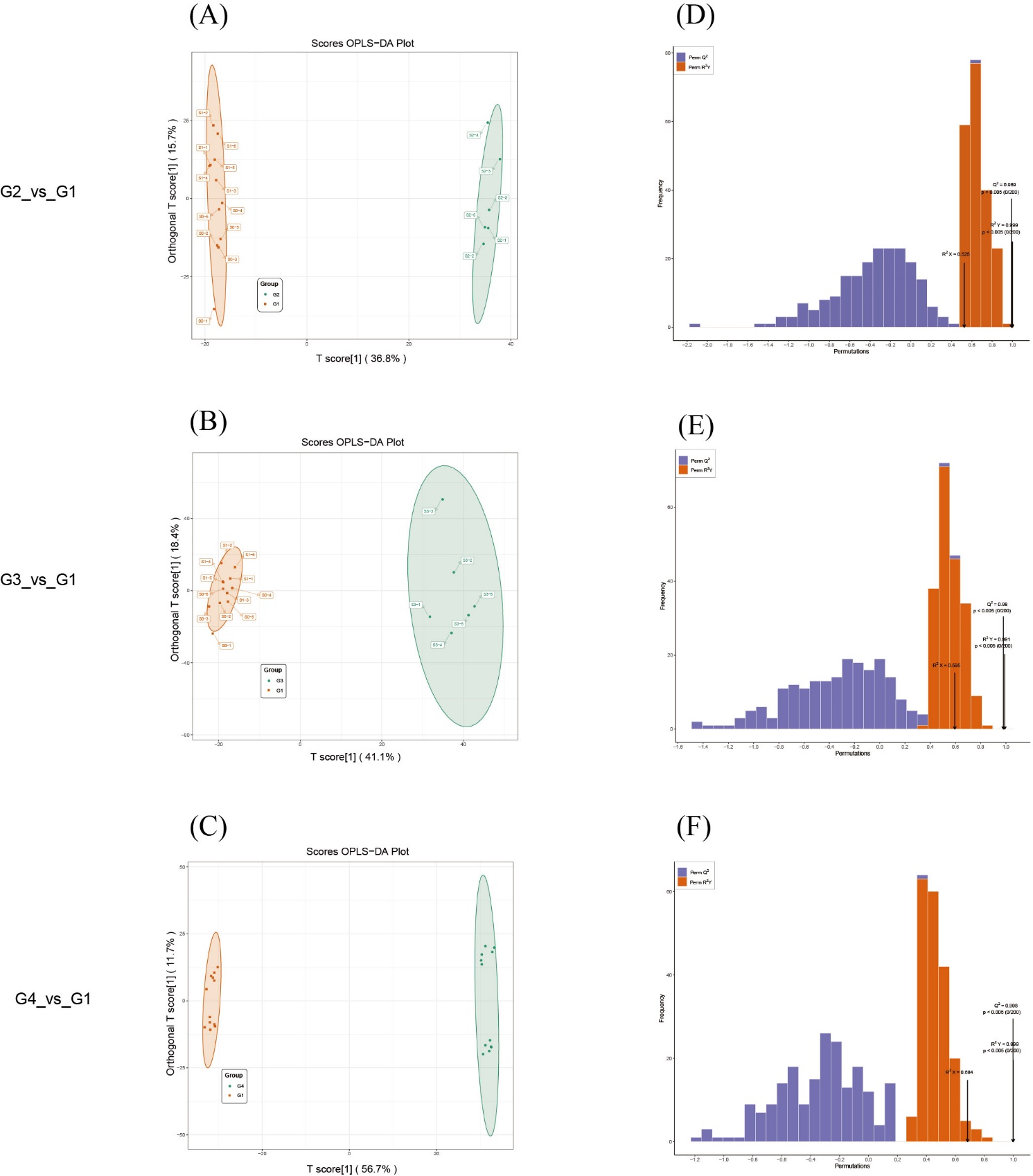
**

**Supplementary Figure 1.** OPLS-DA score plots and permutation test validation for different cigar aging stages.

A-C, OPLS-DA score plots of G1 vs G2, G1 vs G3, and G1 vs G4respectively. D-F, represent results of 200-permutation tests for OPLS-DA models: G1 vs G2, G1 vs G3, G1 vs G4, respectively.


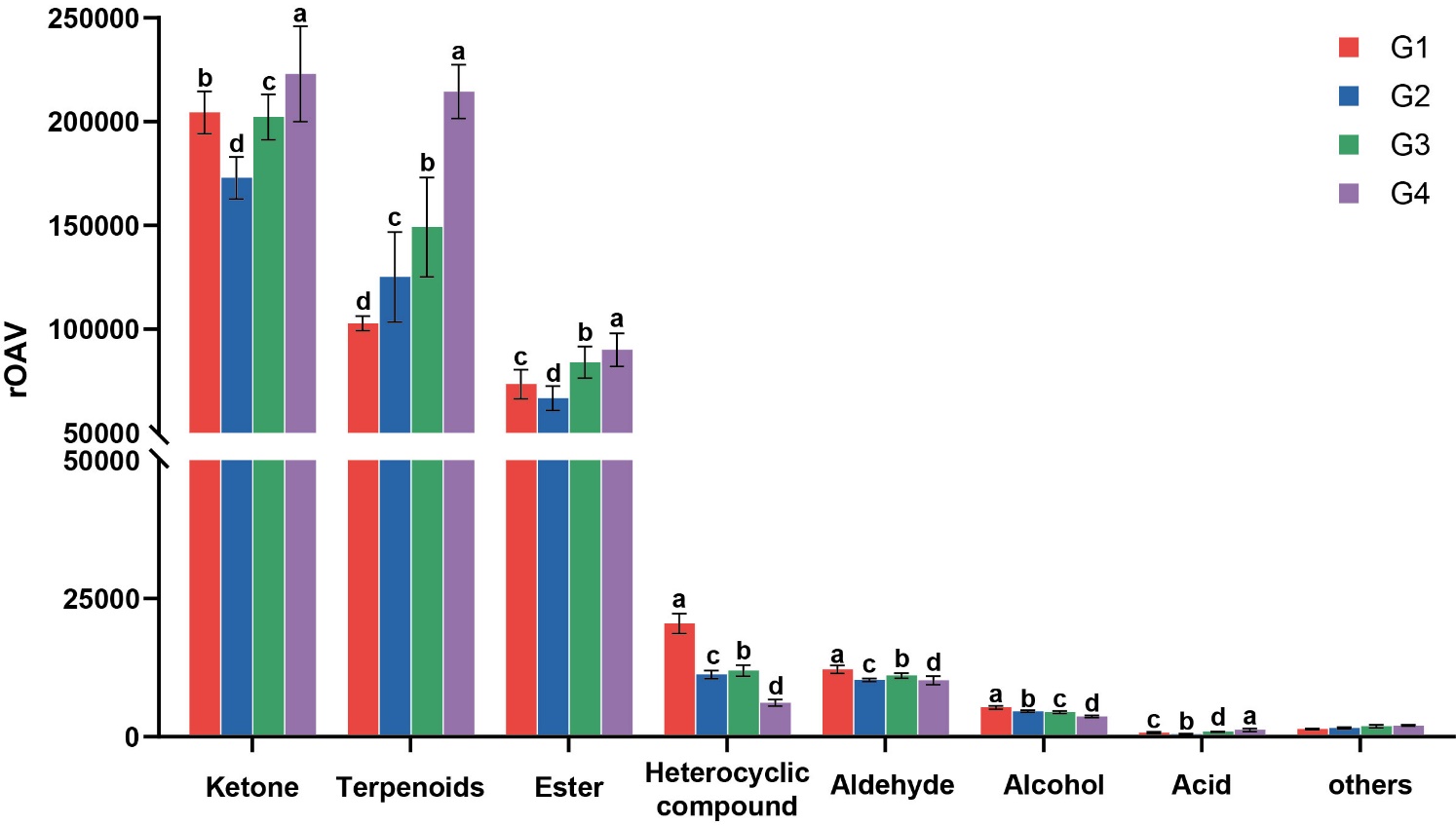


**Supplementary Figure 2.** Changes in rOAV values of different classes of volatile metabolites during cigar aging. Different letters indicate significant differences among groups (*p* < 0.05).
